# Reductive metabolism of the important atmospheric gas isoprene by homoacetogens

**DOI:** 10.1038/s41396-018-0338-z

**Published:** 2019-01-14

**Authors:** Miriam Kronen, Matthew Lee, Zackary L. Jones, Michael J. Manefield

**Affiliations:** UNSW Water Research Centre, School of Civil and Environmental Engineering, UNSW Australia, Sydney, NSW 2052 Australia

**Keywords:** Biogeochemistry, Environmental sciences

## Abstract

Isoprene is the most abundant biogenic volatile organic compound (BVOC) in the Earth’s atmosphere and plays important roles in atmospheric chemistry. Despite this, little is known about microbiological processes serving as a terrestrial sink for isoprene. While aerobic isoprene degrading bacteria have been identified, there are no known anaerobic, isoprene-metabolizing organisms. In this study an H_2_-consuming homoacetogenic enrichment was shown to utilize 1.6 μmoles isoprene h^−1^ as an electron acceptor in addition to HCO_3_^−^. The isoprene-reducing community was dominated by *Acetobacterium* spp. and isoprene was shown to be stoichiometrically reduced to three methylbutene isomers (2-methyl-1-butene (>97%), 3-methyl-1-butene (≤2%), 2-methyl-2-butene (≤1%). In the presence of isoprene, 40% less acetate was formed suggesting that isoprene reduction is coupled to energy conservation in *Acetobacterium* spp. This study improves our understanding of linkages and feedbacks between biogeochemistry and terrestrial microbial activity.

## Introduction

Ecosystems emit numerous biogenic volatile organic compounds (BVOCs), which affect atmospheric chemistry and therefore the Earth’s climate [1–4]. By mass, between 30 and 50% of the estimated total global BVOC flux is isoprene (2-methyl-1,3-butadiene (CH_2_ = C(CH_3_)-CH = CH_2_)), a highly volatile, unsaturated hydrocarbon [5, 6]. An annual global terrestrial isoprene emission of 500–600 Tg per year [7, 8] and an oceanic emission of 0.1–1.2 Tg per year [9] has been estimated, which is similar in magnitude to methane sources at 526–569 Tg per year [10]. The physiological role of isoprene in natural environments remains enigmatic [11]. It is mainly emitted by woody plants [12–15], though it is also released in the breath of humans and other mammals [16, 17], marine algae [18], and by Gram-positive (e.g., *Bacillus* species) and Gram-negative bacteria (e.g., *Escherichia coli*, *Pseudomonas fluorescens*, various actinomycetes *Pseudonocardia*, *Saccharomonospora*, *Streptomyces*, *Thermomonospora*) [19–21].

In the atmosphere, isoprene reaction products modulate the oxidizing potential, which leads to a prolonged lifetime of greenhouse gases such as methane [12, 22–24]. Additionally, isoprene oxidation products cause secondary organic aerosols (SOA) formation [25], which affects the climate directly by scattering absorbance and indirectly via nuclei cloud formation [26, 27].

While sources of atmospheric isoprene have been well-studied, terrestrial fates of isoprene remain relatively unexplored. Soils containing isoprene degrading microorganisms could serve as an important sink as shown by Cleveland and Yavitt [28]. They estimated the global soil isoprene sink at 20.4 Tg per year, which is 4% of the estimated annual biogenic emission of isoprene [5]. Under aerobic conditions, most soil bacteria that have been shown to utilize isoprene as their sole carbon and energy source belong to the phylum *Actinobacteria* [28–31]. The most detailed biochemical characterization of an aerobic isoprene-metabolizing organism was conducted on *Rhodococcus* sp. strain AD45 [31–35]. These studies identified isoprene epoxide (1,2-epoxy-2-methyl-3-butene) and the two glutathione adducts, 1-hydroxy-2-glutathionyl-2-methyl-3-butene (HGMB) and 2-glutathionyl-2-methyl-3-butenoic acid (GMBA), as intermediates of isoprene oxidation [31–33]. Crombie et al. [34] published the whole genome of *Rhodococcus* sp. AD45 and identified additional genes involved in isoprene metabolism, though the complete pathway has not been resolved.

In the present study, the fate of isoprene in anoxic environments was investigated for the first time. Given the environmental abundance and ubiquity of isoprene, we hypothesized that it is available to anaerobic microorganisms. Samples taken from different environments were used to enrich for anaerobic isoprene utilizing microorganisms with the aim of determining the fate of isoprene in anaerobic microbial systems in order to better understand its global degradation pathways. We show for the first time that obligate anaerobes also transform isoprene. Moreover, whereas isoprene primarily serves as an electron donor in aerated soils, we provide evidence that is used as an electron acceptor to support homoacetogenesis.

## Materials and methods

### Chemicals

Isoprene 99% stock solution, 3-methyl-1-butene ≥ 99.0% (GC), 2-methyl-2-butene ≥ 99.0% (GC) and 2-methyl-1-butene ≥ 99.5% (GC), ethylene 99.9% in steel cylinder were all purchased from Sigma-Aldrich, Castle Hill, Australia. Helium (>99.9999% purity), nitrogen gas (>99.99% purity), and air (zero grade purity) were purchased from BOC Gas, Australia. H_2_ (>99.99995% purity) was obtained from a H_2_ generator (Parker domnick hunter, UK).

### Inocula

Sewage sludge samples were obtained from St. Marys Sewage Treatment Plant Sydney, Australia and stored anaerobically in the dark at 4 °C. Soil samples from Botany Bay, Sydney, Australia were core drilled from 3.6 meters beneath the surface and stored in the dark at room temperature in anaerobic media. Soil samples from 5 cm beneath the surface of Colo River area in Wollemi National Park, Blue Mountains Australia, were stored in the dark at room temperature.

### Microbial strains

*Acetobacterium* species *A. woodii* DSM 1030, *A. malicum* DSM 4132, and *A. wieringae* DSM 1911 were obtained from Deutsche Sammlung von Mikroorganismen und Zellkulturen (DSMZ, Germany).

### Culture conditions

Cells were grown in minimal media containing NH_4_Cl (1.2 g l^−1^), MgCl_2_∙ 2H_2_O (0.4 g l^−1^), and CaCl_2_ ∙ 2H_2_O (0.1 g l^−1^). The media was dispensed into culture flasks, flushed with nitrogen for 20 min, crimp-sealed with Teflon faced rubber septa and autoclaved. After autoclaving trace element mixture (HCl (25%), 10 ml; FeCl_2_ ∙ 4H_2_O, 2150 mg; MnCl_2_ ∙ 4H_2_O, 30 mg; CoCl_2_ ∙ 6H_2_O, 50 mg; CuCl_2_ ∙ 2H_2_O, 34 mg; NiCl_2_ ∙ 6H_2_O, 20 mg; Na_2_MoO_2_ ∙ 2H_2_O, 30 mg; ZnSO_4_ ∙ 7H_2_O, 24 mg; H_3_BO_3_, 20 mg; distilled water, 1 l), 1 ml vitamin solution (niacin, 100 mg; thiamine hydrochloride, 100 mg; biotin, 40 mg; pyridoxol hydrochlorid, 100 mg; folic acid, 20 mg; riboflavin, 50 mg; lipoic acid, 50 mg; pantothenic acid, 50 mg; Vitamin B12, 7.8 mg; 4-aminobenzoic acid, 50 mg; distilled water, 1 l), and 12.5 ml of 1M phosphate buffer (prepared from 1M K_2_HPO_4_ solution by adjusting pH value to 7.0 with 1 M NaH_2_PO_4_) were added aseptically per liter of media. Vitamins were filter sterilized and all other solutions were autoclaved. Cultures were incubated at 25 °C in the dark.

### Initial enrichment cultures for isoprene reduction

Sludge (50 µl, 2 × 10^9 ^cells ml^–1^) was added to sealed culture flasks (120 ml) containing 80 ml anaerobic minimal media. d/l-Lactate was supplied at ~10 mM from a sterile stock solution and isoprene was added as an electron acceptor from a 99.9% stock solution by using a 100 µl glass syringe to a final concentration of 1.3 mM in liquid media. Dilution to extinction series (10^−1^–10^−6^) were performed using the same culture condition except in 60 ml flask containing 40 ml anaerobic minimal media.

### Growth in H_2_ and HCO_3_^−^ containing media

Anaerobic culture flask (120 ml) containing 80 ml minimal media were supplied with 0.5 bar sterile filtered H_2_ and 30 mM NaHCO_3_. Isoprene was added from a 99.9% stock solution by using a 100 µl glass syringe to a final concentration of 1.3 mM in liquid media. In the case of ethylene it was added from a 99.5% gas stock via a 5 ml and 0.5 ml gas tight syringe to 0.38 or 0.02 mM in liquid media. Cultures were inoculated with isoprene reducing enrichment culture (1 ml). In case of fructose it was added from a 1 M anaerobic stock solution to a final concentration of 20 mM.

### Standards

Standards were prepared in 120 ml flasks with 80 ml anaerobic minimal medium. Isoprene was added from stock solution with a glass syringe to prepare standards reaching from 0.4 to 5 mM nominal concentrations. 3-methyl-1-butene, 2-methyl-2-butene, and 2-methyl-1-butene were added from each stock solution with a glass syringe and combined in one flask as a standard reaching from 0.4 to 5 mM nominal concentrations. H_2_ was added from a H_2_ generator with standards reaching from 0.2 to 20 mM nominal concentrations. Ethane, ethylene, and methane standards were prepared from a gas mixture (33.33% gas each) to prepare standards reaching from 0.1 to 12 mM nominal concentrations. All gases were added with different sizes of gas tight syringes. Dimensionless Henry constants for isoprene, ethylene, ethane, methylbutenes, H_2,_ and methane were calculated from Sander [36].

### Isoprene, H_2_, HCO_3_^−^, and hydrocarbon analysis

Isoprene, methylbutenes, methane, H_2,_ CO_2_ and hydrocarbon gases were monitored by gas chromatography (GC) using a Shimadzu GC-2010. Isoprene, methylbutenes and methane were analyzed by using a GasPro PLOT column (60 m x 0.32 mm, Agilent Technologies) with Helium as a carrier gas (3 ml min^−1^) and flame ionization detection (FID). The oven temperature was 150 °C for 30 s and was increased by 20 °C min^−1^ to a final temperature of 250 °C. Gas samples (100 µl) were withdrawn from the flask via a pressure-lockable gas tight syringe and directly injected into the GC.

H_2_ was analyzed using a HP-PLOT Molesieve column (30 m × 0.32 mm × 0.25 mm, Agilent Technologies, Australia) and pulsed discharged ionization detector (PDD). The carrier gas was Helium (3 ml min^−1^) and the oven temperature was applied at 50 °C for 1.2 min. Gas samples (20 µl) were withdrawn from the flask via a gas lock syringe and directly injected into the Shimadzu GC-2010.

HCO_3_^−^ concentrations were measured by acidification of media (100 µl) in a sealed flask resulting in the release of HCO_3_^−^ in form of CO_2_. CO_2_ was measured by GC-PDD using a HP-PLOT Q column (30 m x 0.32 mm, Agilent Technologies, Australia). The carrier gas was Helium (3 ml min^−1^) and the oven temperature was applied from 50 °C for 1 min to 54.5 °C with a rate of 3.5 °C. Gas samples (40 µl) were withdrawn from the flask via a gas lock syringe and directly injected into a Shimadzu GC-2010.

Formate, acetate, butyrate, and propionate were analyzed as their ethyl ester derivative by GC-FID using a DB-FFAP column (30 m x 0.32 mm × 0.25 mm, Agilent Technologies) at 40 °C for 6 min with helium as the carrier gas (3 ml min^−1^). Samples (500 µl media) were supplied with 100% ethanol (200 µl) and undiluted sulfuric acid (200 µl) for esterification, sealed immediately and incubated at 60 °C for 45 min. Before injection into the GC samples were incubated at 80 °C for 5 min at 500 rpm, 250 µl of headspace sample was withdrawn from the flask via an automatic sampler (Shimadzu AOC-5000 plus) and directly injected into a Shimadzu Plus GC-2010 at 500 µl s^−1^.

Ethene and ethane amounts were measured by GC-FID using a GS-Q column (30 m x 0.32 mm × 0.25 mm) at 100 °C for 2 min with helium as the carrier gas (3 ml min^−1^). Gas samples (100 µl) were withdrawn from the flask via a gas lock syringe and directly injected into a Shimadzu GC-2010.

### D/L-Lactate analysis

Lactate concentrations were monitored by using the d/l-lactic acid kit from Megazyme following manufacturer’s instructions.

### DNA extraction and Illumina sequencing

DNA was extracted from 300 µl culture using standard phenol–chloroform extraction method. Lysis buffer [37] was added to the sample and the tube was mechanically agitated in FastPrep Lysis Matrix A tubes (MP Biomedicals). DNA was extracted with sequential phenol (phenol–chloroform–isoamyl alcohol (25:24:1), 7.5 M ammonium acetate, chloroform and isopropanol treatments, precipitated with ethanol using a general protocol, resuspended in 20 µl H_2_O and stored at −20 °C until further analysis. Regions of 16S rDNA gene were amplified by PCR from extracted DNA with the Q5 high-fidelity DNA polymerase (New England BioLabs) using the universal primers 926F (5′-TCGTCGGCAGCGTCAGATGTGTATAAGAGACAG -[AAA CTYAAAKGAATTGRCGG]-3′) and 1392R (5′GTCTCGTGGGCTCGGAGATGTGTATAAGAGACAG -[ACG GGC GGT GTG TRC-3′) targeting bacteria and archaea [38]. The samples were sequenced on an Illumina MiSeq Sequencer (Illumina, USA) using V3 chemistry at the Next Generation Sequencing Facility at Western Sydney University’s Hawkesbury Institute for the Environment (Sydney, Australia). In all, 16S rRNA gene amplicon sequences were analyzed with QIIME2 (https://qiime2.org) [39] utilizing the dada2 pipeline [40]. Sequencing quality was first visualized with FastQC (www.bioinformatics.babraham.ac.uk) resulting in forwards and reverse reads being trimmed at 290 base pairs and 240 base pairs, respectively. Forward and reverse sequences that passed the default quality control were merged and non-overlapping sequences were discarded. Chimeras were analyzed and removed via the consensus method within the dada2 pipeline. Remaining sequences had taxonomy assigned with the RDP classifier [41] using the Greengenes 13_8 database [42]. Taxa present at <2% abundance were removed for clarity.

### Cloning

In all, 16S rDNA gene fragment cloning was performed on DNA samples from the isoprene reducing culture after 29 days [Fig. 7]. Part of the *Acetobacterium* 16S rDNA fragment was PCR-amplified with the Aceto572F and Aceto784R [43] and a constant annealing temperature of 59 °C for 34 cycles. All products were cloned into the pCR™2.1-TOPO® vector with TOPO TA Cloning Kit (Invitrogen, Carlsbad, CA) according to manufacturer's instructions. Plasmid DNA was extracted from overnight *Escherichia coli* (DH5α™-T1®) cultures using the PureYield™ Plasmid Miniprep System (Promega, Fitchburg, WI).

### Quantitative real-time PCR

Quantitative real-time PCR targeting *Acetobacterium* was performed on a Biorad real-time PCR system by using QuantiTect SYBR green PCR mastermix (Qiagen, Germany) and *Acetobacterium* primers Aceto572F and Aceto784R. The thermocycling program was as follows: initial denaturation for 3 min at 98 °C; 39 cycles of [95 °C for 20 s, 59 °C annealing for 50 s] and a final melting curve analysis from 60 to 99 °C. The standard curve was generated with serial dilutions of a known quantity of 16S rDNA *Acetobacterium* gene-contained in plasmids generated as described above. Five copies of 16S rRNA genes per *Acetobacterium* genome were calculated based on the sequenced genome of *A. woodii* DSM 1030 to convert gene copies to cell numbers [44].

## Results

### Isoprene transformation in anaerobic microcosms

Isoprene did not serve as an electron donor for the reduction of SO_4_^2−^_,_ NO_3_^2−^, and Fe^3+^ after 12 months of observation in any of the tested inocula (data not shown). However, the evolution of isoprene reduction products 2-methyl-1-butene (>97%), 3-methyl-1-butene (≤2%), and 2-methyl-2-butene (≤1%) was observed in activated sludge microcosms, suggesting the compound is reduced by the inocula [Mass spectra: Supplementary Fig. S1]). To investigate the reduction of isoprene more closely quadruplicate anaerobic microcosms were prepared with lactate (900 μmoles) as the carbon and energy source, isoprene (160 μmoles) as the electron acceptor, and activated sludge as inoculum (1.7 × 10^7 ^cells ml^–1^ final concentration).

**Fig. 1:**
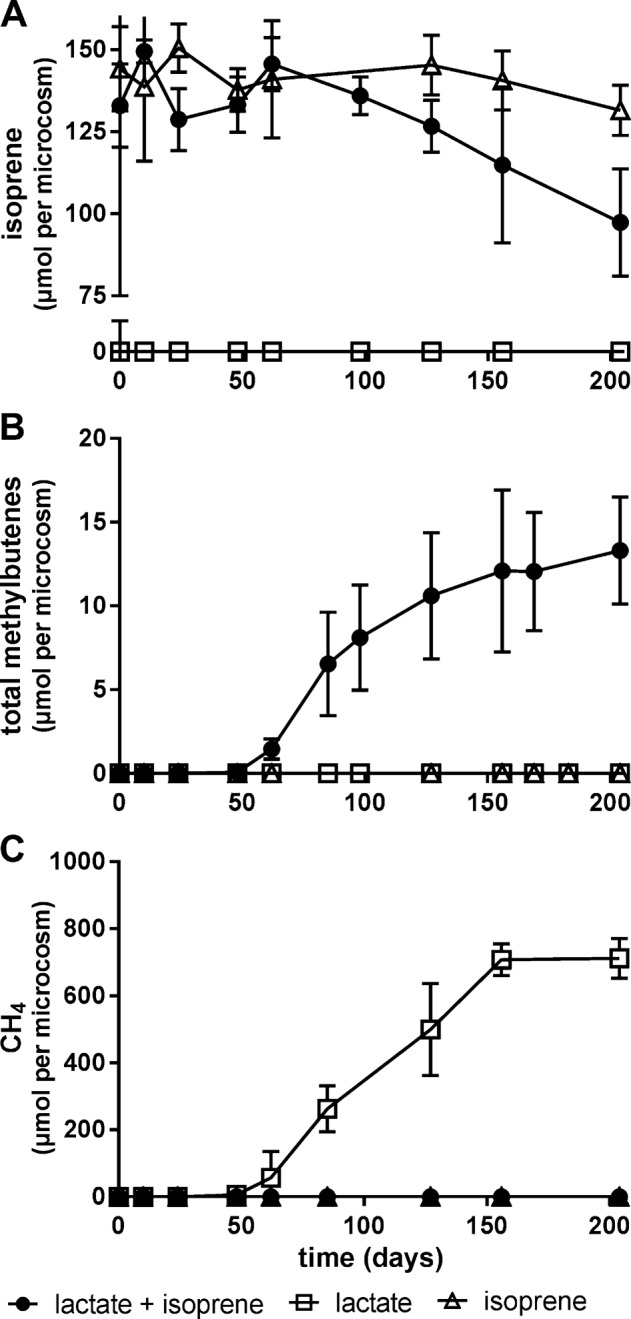
Depletion of isoprene (**a**) and subsequent production of methylbutenes (**b**) was only observed in microcosms containing sludge, lactate, and isoprene. In control samples supplemented with only lactate or isoprene, no isoprene depletion or methylbutene formation was detected. Methane (**c**) production only occurred in cultures supplied with lactate. Error bars represent one standard deviation (*n* = 4)

**Fig. 2:**
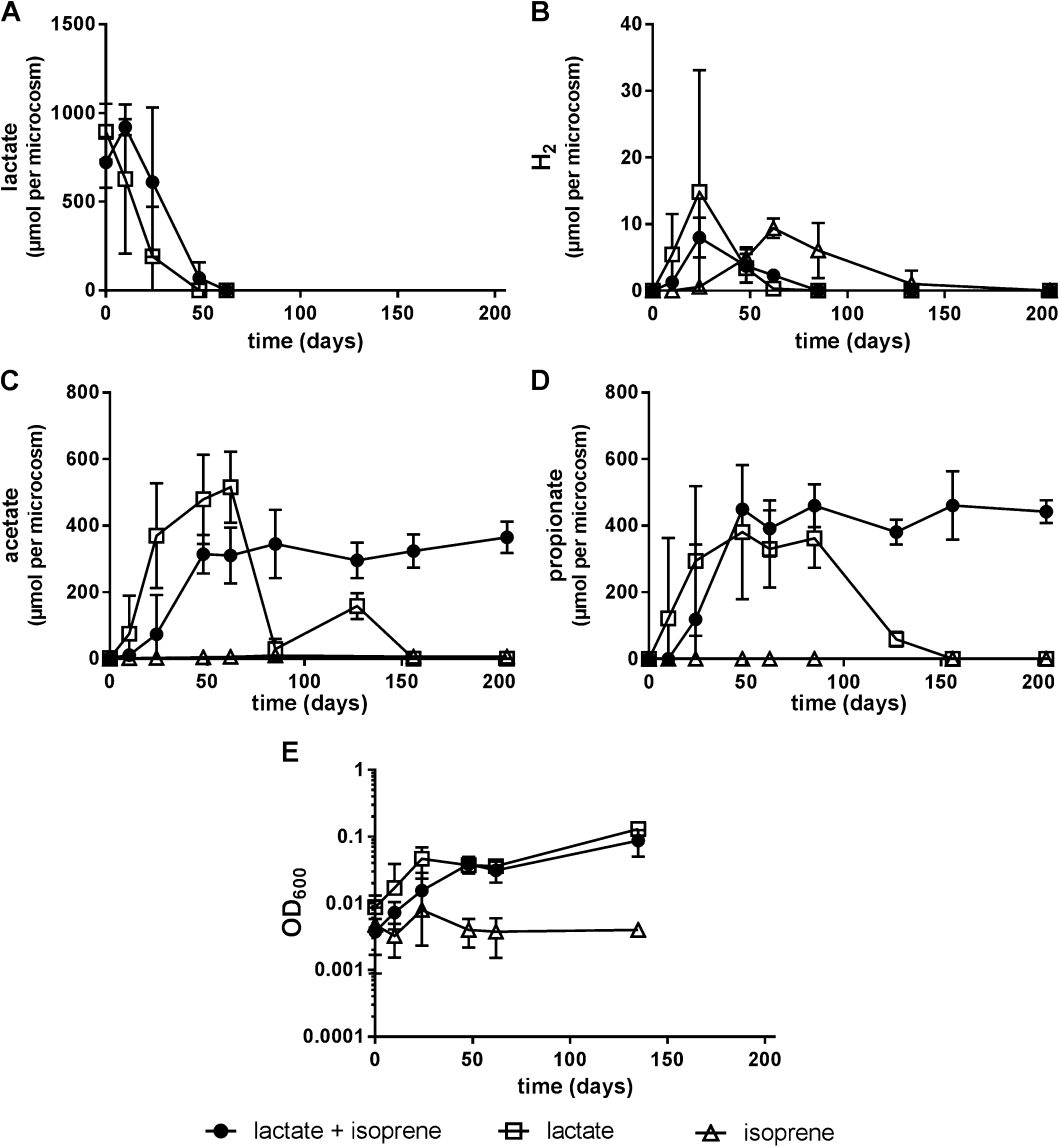
Change in quantity of lactate (**a**), H_2_ (**b**), acetate (**c**), propionate (**d**), and biomass formation (**e**) in anaerobic cultures containing sludge and amended with lactate, isoprene, or both. Acetate and propionate were depleted in cultures with lactate only but concentrations remained stable after day 200 in cultures with isoprene and lactate. Error bars represent one standard deviation (*n* = 4)

In the first round of enrichment cultures, isoprene depleted at a rate of ~0.3 μmoles per day after a 50 day lag period, with concomitant production of 15 μmoles methylbutenes after 200 days [Fig. 1a, b]. The predominant isoprene reduction product was 2-methyl-1-butene (98%, 12.3 μmoles), with lesser amounts of 3-methyl-1-butene (2%). Lactate (900 μmoles) was completely consumed after 50 days in the presence or absence of isoprene [Fig. 2a] and was fermented to acetate, propionate (400 μmoles each), H_2_ with associated biomass generation [Fig. 2b–e]. Isoprene reduction occurred subsequent to lactate consumption (after 50 days). Cultures without lactate amendment generated low quantities of fatty acids (<6 μmoles) and H_2_ (~3−10 μmoles) presumably derived from biomass supplied in the inoculum, but did not convert isoprene to methylbutenes. Methane production was not detected in the presence of isoprene and lactate, however in the presence of lactate alone methane production commenced after 48 days and increased to 700 μmoles within 100 days with associated depletion of acetate and propionate [Figs. 1c and 2c, d]. Autoclaved and uninoculated controls did not show any depletion of isoprene or formation of methylbutenes.

### Community analysis of lactate driven anaerobic isoprene transformations

**Fig. 3:**
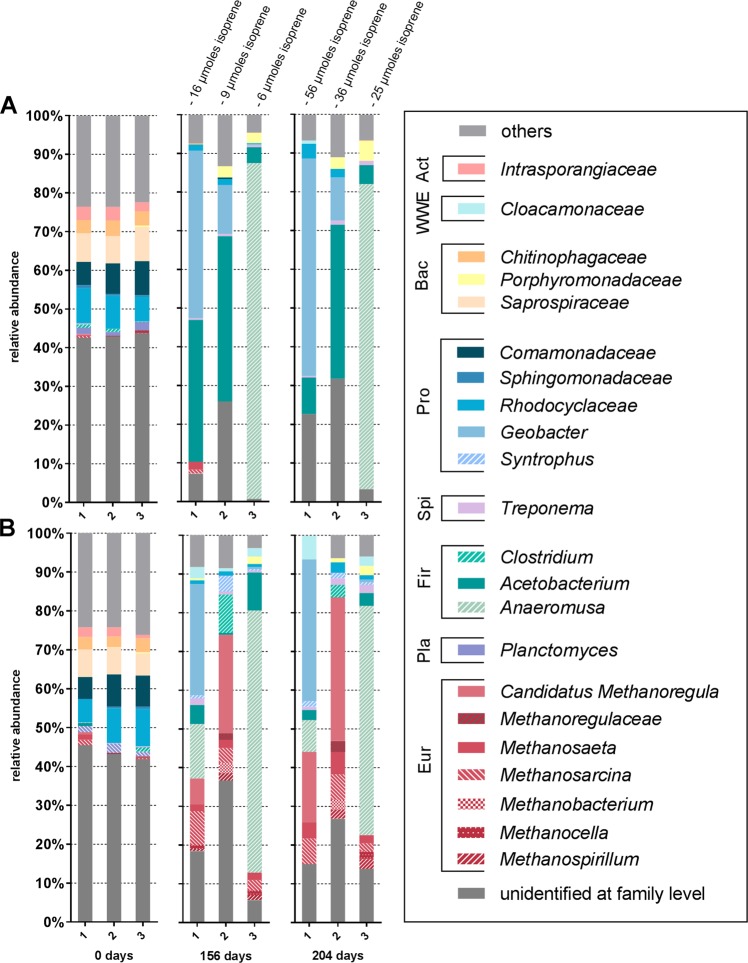
Composition of the bacterial and archaeal populations classified at Family level and if possible on Genus level of sludge during growth on lactate + isoprene (**a**) and lactate only control (**b**) at time points 0, 156, and 204 days. Samples were inoculated with wastewater from St. Marys treatment plant. Other category is the sum of all classifications with <2% abundance. Classifications in the legend are clustered according to their phylum (from top to bottom); *Actinobacteria*, *WWE1*, *Bacteroidetes*, *Proteobacteria*, *Spirochaetes*, *Firmicutes*, *Planctomycetes*, *Euryarchaeota*. Depleted amounts of isoprene in each replicate are shown at the top. Only three out of four replicates were analyzed by Illumina sequencing

Bacterial and archaeal community analysis was performed using 16S rRNA gene amplicons derived from DNA extracted from the lactate and isoprene fed cultures sampled before and after isoprene consumption [Fig. 3]. In the isoprene fed cultures, *Acetobacterium*, *Geobacter*, and in one replicate *Anaeromusa* where found to be the dominant bacterial genera [Fig. 3a]. Cultures supplied with lactate but without isoprene were dominated by a consortium of methanogenic archaea and bacteria *Geobacter*, *Clostridium*, *Acetobacterium*, and *Anaeromusa* [Fig. 3b].

### Characterization of H_2_ driven isoprene transformations

The lag in isoprene reduction relative to lactate depletion suggested that lactate fermentation products were serving as carbon and energy sources. Given that acetate and propionate concentrations remained stable after day 200 in cultures amended with isoprene [Fig. 2c, d] it was hypothesized that H_2_ and HCO_3_^−^ were serving as electron donor and carbon source, respectively.

To test this hypothesis a dilution to extinction experiment was set-up with H_2_ as the electron donor, HCO_3_^−^ as the carbon source, and isoprene as the electron acceptor using a pooled enrichment culture derived from lactate and isoprene amended cultures as an inoculum. Isoprene reduction was observed in the 10^−6^ dilution with isoprene quantitatively transformed to 2-methyl-1-butene (98%) at a rate of 1.6 μmoles h^−1^.

**Fig. 4:**
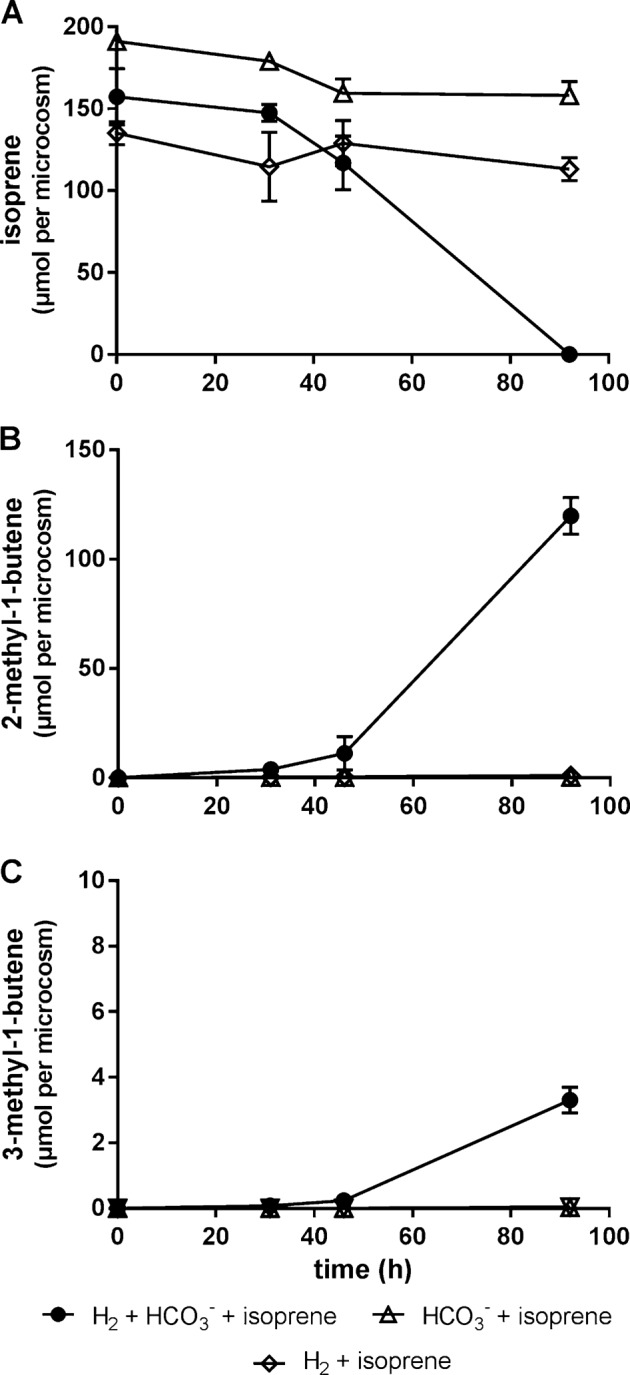
Depletion of isoprene (**a**) in 80 ml anaerobic cultures containing an enriched isoprene reducing culture supplied with H_2_ + HCO_3_^−^ + isoprene and reciprocal production of 2-methyl-1-butene (**b**) and 3-methyl-1-butene (**c**). In control samples supplemented with only H_2_ + isoprene or only HCO_3_^−^ + isoprene no methylbutene formation or isoprene depletion was detected. Error bars represent one standard deviation (*n* = 4)

**Fig. 5:**
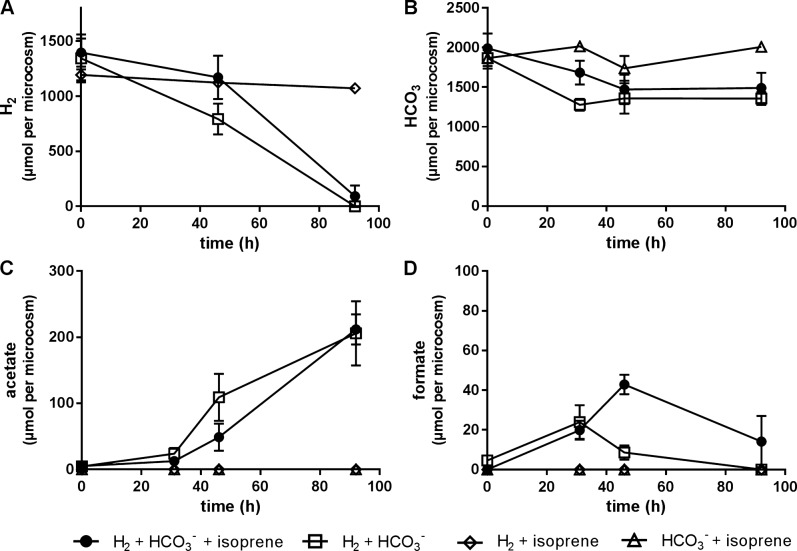
Consumption of H_2_ (**a**) and HCO_3_^−^ (**b**) and formation of acetate (**c**) and formate (**d**) anaerobic cultures containing an enriched isoprene reducing culture supplied with and without isoprene. In control samples supplemented with H_2_ + isoprene or HCO_3_^−^ + isoprene no acetate or formate formation was detected. Error bars represent one standard deviation (*n* = 4)

Having established the defined conditions for the cultivation of isoprene reducing bacteria (i.e., H_2_/HCO_3_^−^/isoprene), quadruplicate cultures were prepared to characterize H_2_/HCO_3_^−^/isoprene consumption rates and formation of volatile fatty acids (i.e., acetate, and formate). Isoprene (130 μmoles) was transformed to methylbutenes (125 μmoles) within 92 h [Fig. 4a–c]. The predominant methylbutene was 2-methyl-1-butene (97%) with lesser amounts to 3-methyl-1-butene (2%), and 2-methyl-2-butene (≤1%) [Fig. 4b, c]. Depletion of H_2_ and HCO_3_^−^ correlated with an increase in acetate. There was no significant difference in H_2_ and HCO_3_^−^ depletion or acetate and formate production when comparing isoprene and isoprene free cultures [Fig. 5a–d]. When cultures were incubated with isoprene and H_2_ or isoprene and HCO_3_, neither acetate formation nor isoprene reduction was observed. To determine if methylbutene can be further reduced, H_2_ was resupplied. After 8 days no further reduction of methylbutene to methylbutane was observed [Supplementary Fig. S2].

### Community analysis of H_2_ driven anaerobic isoprene transformations

**Fig. 6:**
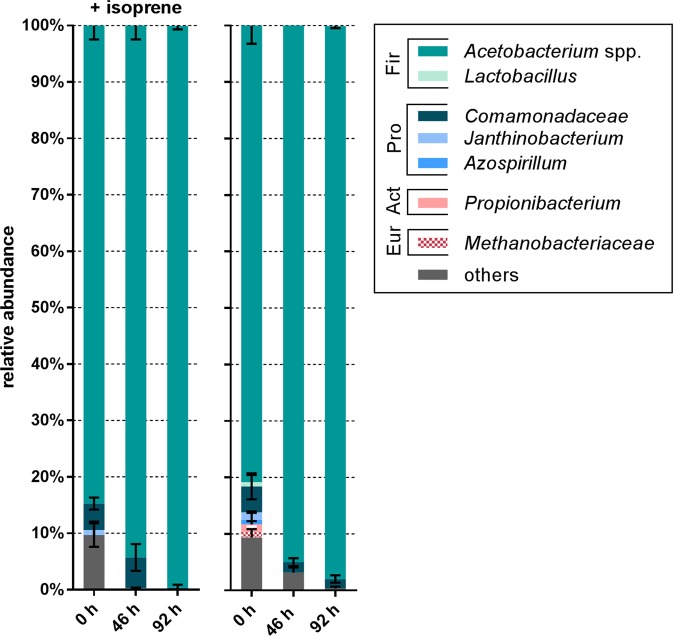
Composition of the bacterial populations at Family level and if possible on Genus level of the isoprene reducing culture during growth on H_2_ + HCO_3_^−^ + isoprene and on H_2_ + HCO_3_^−^ controls at different time points 0, 46, and 96 h. Other category is the sum of all classifications with <2% abundance. Error bars represent one standard deviation (*n* = 3). Classifications in the legend are clustered according to their phylum (from top to bottom); *Firmicutes*, *Proteobacteria*, *Actinobacteria*, *Euryarchaeota*

Illumina sequencing of 16S rRNA gene amplicons from H_2_ supplied, isoprene reducing enrichment cultures revealed enrichment of *Acetobacterium* to 92–100% relative abundance [Figs. 4a and 6]. *Comamonadaceae* were also present (2–7%). There was no notable difference between bacterial community compositions in the presence vs. absence of isoprene [Fig. 6].

**Fig. 7:**
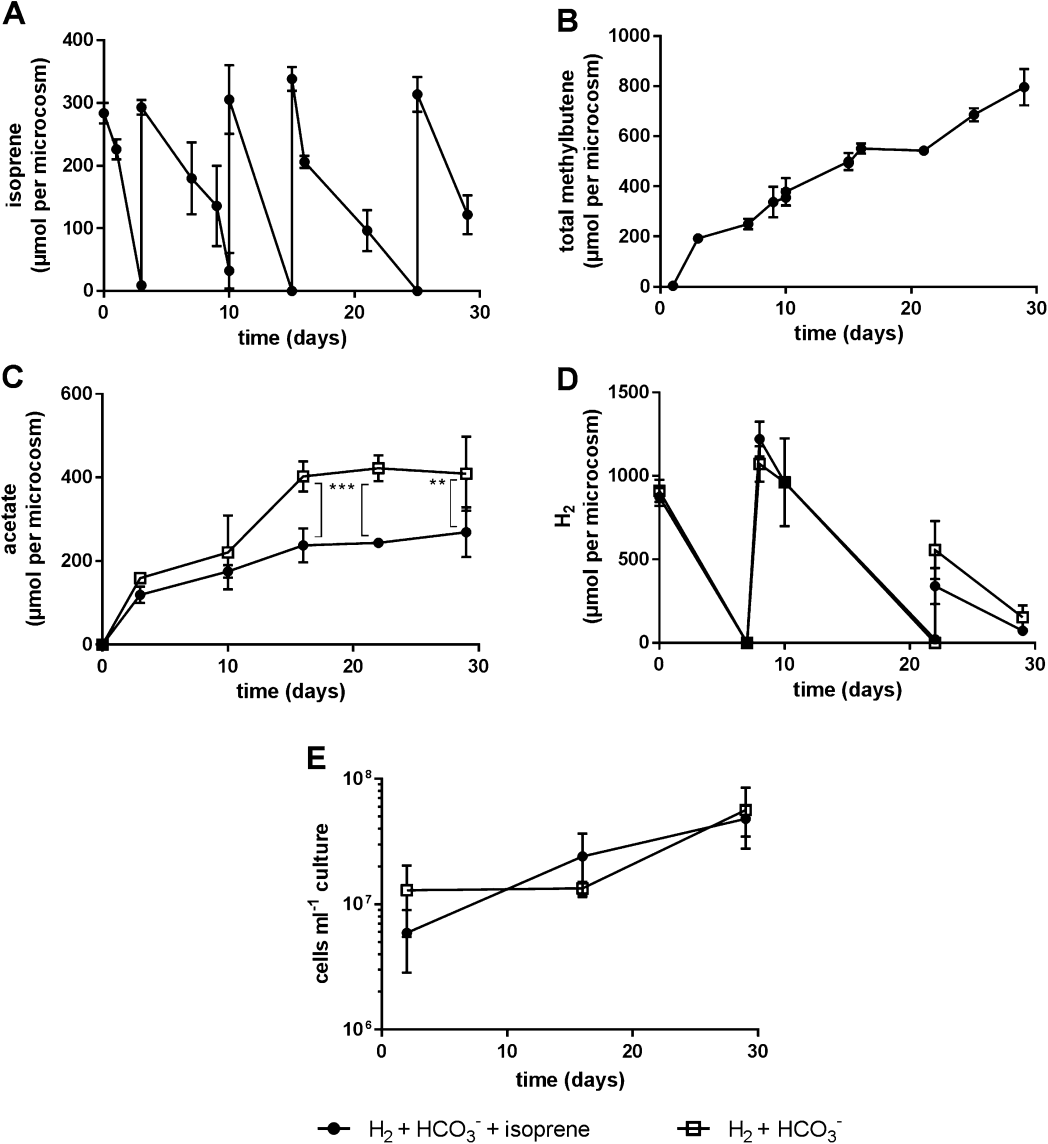
Depletion of isoprene (**a**) and reciprocal production of total methylbutenes (**b**) in anaerobic cultures containing an enriched isoprene reducing culture amended with H_2_ and HCO_3_^−^ with and without isoprene. Cultures amended with isoprene produced 40% less acetate (**c**) while still consuming the same amount of H_2_ (**d**). *Acetobacterium* cells ml^−1^ (**e**) calculated from 16S rDNA gene copies ml^−1^ demonstrate equivalent growth in both conditions. ***p-value < 0.0001, **p-value = 0.005 analyzed by a two-way ANOVA. Error bars represent one standard deviation (*n* = 4)

To further probe an isoprene dependent difference in community composition a new set of cultures was monitored over a longer period by resupply of isoprene, H_2_, and HCO_3_^−^ when depleted [Fig. 7]. Isoprene was depleted within 3–5 days after each resupply at a similar rate as observed before (1.6 μmoles h^−1^) [Fig. 7a]. Methylbutene accumulated up to 800 μmoles, respectively [Fig. 7b]. Illumina sequencing of 16S rRNA amplicons again showed no appreciable difference in microbial community composition when comparing cultures with or without isoprene. Both communities were once again dominated by *Acetobacterium* spp. [Supplementary Fig. S3]. Additionally, *Acetobacterium* cell numbers were similar after 29 days (i.e., ~4.8 × 10^7^ ± 1.3 × 10^7 ^cells ml^−1^ for cultures with isoprene and 5.6 × 10^7^ ± 2.8 × 10^7 ^cells ml^−1^ for those without) [Fig. 7e]. Importantly, however, there was a significant difference in the amount of acetate produced in cultures with and without isoprene. After day 16, cultures with isoprene produced ~250 μmoles of acetate while those without produced ~400 μmoles [Fig. 7c].

### No transformation of isoprene by pure *Acetobacterium* strains

*A. woodii* DSM 1030, *A. malicum* DSM 4132, and *A. wieringae* DSM 1911 were tested for isoprene reduction on H_2_/HCO_3_^−^/isoprene and fructose/isoprene but showed no isoprene reduction activity.

### No transformation of ethylene by isoprene reducing enrichment culture

To test the substrate specificity of the isoprene reducing culture, ethylene was tested for reduction. Ethylene (C_2_H_4_) contains a single double bond and is another volatile alkene that is emitted by plants.

Quadruplicate anaerobic cultures (80 ml) were prepared with two ethylene concentrations (i.e., 10 or 160 μmoles per flask). H_2_ was supplied as the electron donor. Samples were inoculated with an active isoprene reducing culture (1 ml, 0.8% v/v). Ethylene remained unchanged after 22 days of incubation. Acetogenic growth was not affected by ethylene and cells reduced CO_2_ to around 600 μmoles of acetate [Supplement Figs. S4 and S5].

## Discussion

### Isoprene serves as an electron acceptor

Even though isoprene is a highly abundant, energy rich metabolite, little is known about its anaerobic metabolism. In this study we tested the utility of isoprene as both an electron donor for inorganic oxide reduction and as an electron acceptor where lactate and molecular H_2_ were electron donors.

Anaerobic isoprene oxidation coupled to inorganic oxide reduction (SO_4_^2−^_,_ NO_3_^2−^, or Fe^3+^) could not be demonstrated in any of the tested inocula after 1 year of incubation. Under standard conditions, anaerobic isoprene oxidation is exergonic, considering the theoretical stoichiometries of isoprene mineralization and SO_4_^2−^, Fe^3+^, or NO_3_^−^ reduction (isoprene energy of formation calculated as 197 kJ mol^−1^ [45, 46].

1 $$ {\begin{array}{*{20}{c}} \begin{array}{l}2\,{{{\rm{C}}_5{\rm{H}}_8}} + 2\,{{{\rm{H}}_2{\rm{O}}}} + 7\,{{{\rm{SO}}_4}}^{2 - } \to 7\,{\mathrm{HS}}^ - \\ + 3\,{\mathrm{H}}^ + + 10\,{\mathrm{HCO}}_3^ - \end{array} & {\Delta G^\circ = - 605\,{\mathrm{kJ}}{\mathrm{/}}{\mathrm{mol}^{-1 }}} \end{array}}$$

2 $${\begin{array}{*{20}{c}} \begin{array}{l}{{{\rm{C}}_5{\rm{H}}_8}} + {{{\rm{H}}_2{\rm{O}}}} + 14\,{{{\rm{NO}}_3}}^ - \to 5{{{\rm{HCO}}_3}}^ - \\ + 5\,{\mathrm{H}}^ + + 14\,{{{\rm{NO}}_2}}^ - \end{array} & {\Delta G^\circ = - 2044{\mathrm{kJ}}{\mathrm{/}}{\mathrm{mol}^{-1 }}} \end{array}}$$

3 $${\begin{array}{*{20}{c}} \begin{array}{l}28\,{\mathrm{Fe}}^{3 + }{\mathrm{ + }}{{{\rm{C}}_5{\rm{H}}_8}} + 15\,{{{\rm{H}}_2O}} \to 5\,{{{\rm{HCO}}_3}}^ - \\ + 28\,{\mathrm{Fe}}^{2 + }{\mathrm{ + }}33{\mathrm{H}}^ + \end{array} & {\Delta G^\circ = - 2914\,{\mathrm{kJ}}{\mathrm{/}}{\mathrm{mol}^{-1 }}} \end{array}}$$

Evidently the enzyme systems required for the above transformations are either non-existent, extremely rare or inhibited or not induced under the conditions applied.

**Fig. 8:**
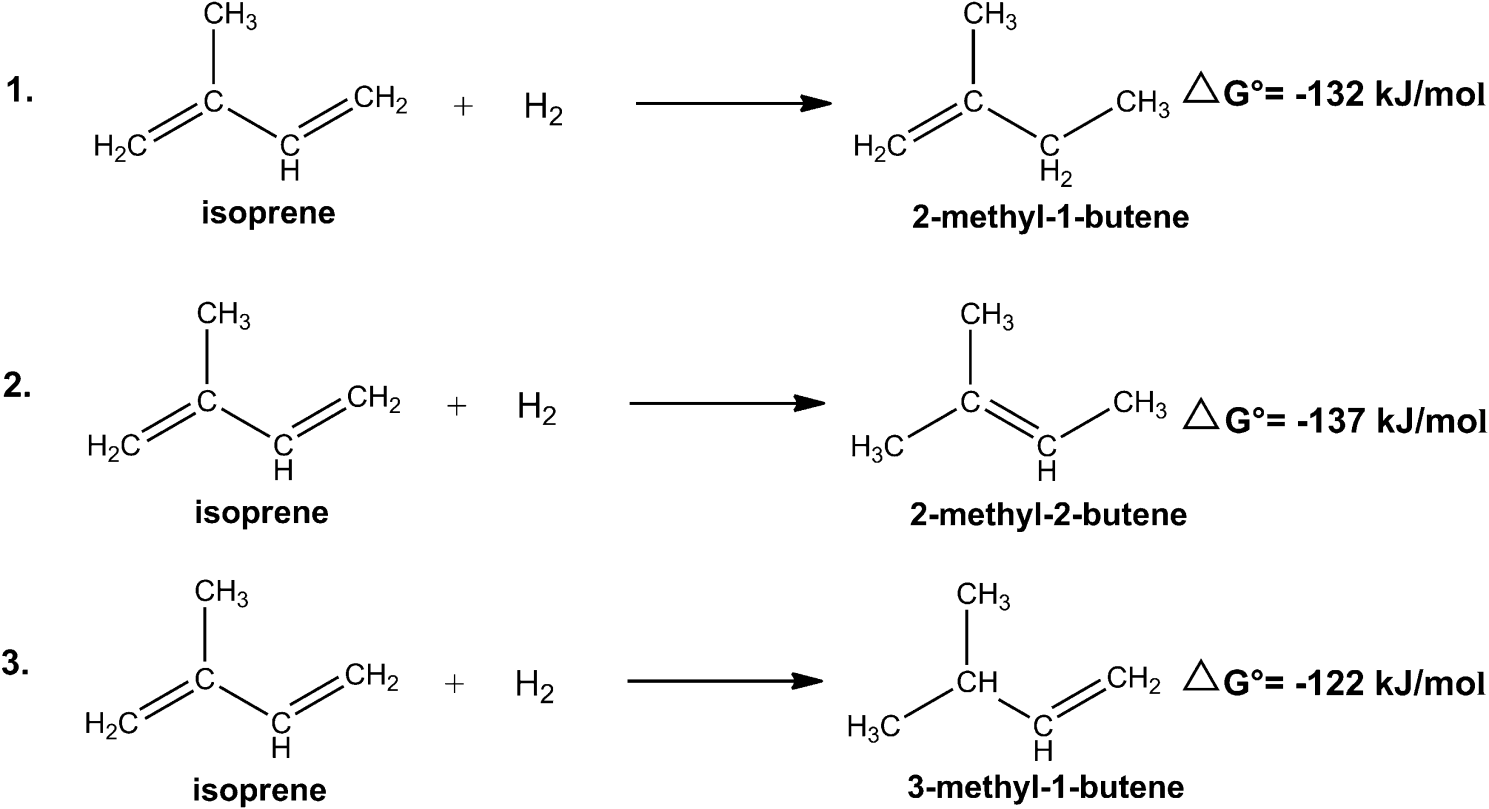
Chemical equations and Gibbs free energy of formation of the reduction of isoprene to 3-methyl-1-butene, 2-methyl-1-butene, and 2-methyl-2-butene with H_2_ as the electron donor. Gibbs free energy of formation of isoprene was estimated by the group contribution method [44] to be 197 kJ mol^–1^

However, reductive isoprene transformation to 2-methyl-1-butene, 3-methyl-1-butene, and 2-methyl-2-butene was observed under methanogenic conditions after 2 months of incubation. Subsequent enrichment resulted in increased rates of isoprene reduction (from ~0.3 μmoles per day to 40 μmoles per day). Isoprene reduction to methylbutene is also thermodynamically favorable considering theoretical stoichiometries with H_2_ as electron donor [Fig. 8] [45, 47].

4 $$\begin{array}{*{20}{c}} {{\mathrm{H}}_2 + {{{\rm{C}}_5{\rm{H}}_8}} \to {{{\rm{C}}_5{\rm{H}}}}_{10}} & {\Delta G^\circ = - 137\,{\mathrm{kJ}}{\mathrm{/}}{\mathrm{mol}^{-1 }}} \end{array}$$

### *Acetobacterium* spp. reduce isoprene

Illumina sequencing targeting archaeal and bacterial 16S rRNA gene amplicons revealed that acetogenic *Acetobacterium* spp. dominated the isoprene reducing enrichment culture. Acetogens are anaerobic bacteria that use CO_2_ as a terminal electron acceptor for energy conservation and carbon fixation utilizing the reductive acetyl coenzyme A (acetyl-CoA) pathway or Wood-Ljungdahl pathway [48, 49]:

5 $${\begin{array}{*{20}{c}} {{\mathrm{CO}}_2 + 4\,{\mathrm{H}}_2 \to {{{\rm{CH}}_3{\rm{COOH}}}} + 2\,{{{\rm{H}}_2{\rm{O}}}}} & {\Delta G^\circ = - 105\,{\mathrm{kJ}}{\mathrm{/}}{\mathrm{mol}^{-1 }}} \end{array}}$$

They are a phylogenetically and metabolically diverse group using a variety of different electron donors and acceptors [50]. Reducing equivalents can be generated from oxidation of H_2_, C_1_-compounds like methanol and formate, sugars, organic acids, and alcohols [51]. Besides CO_2_, acetogens can also use alternative electron acceptors such as acrylate derivatives [52], fumarate [53], nitrate [54], chlorethenes, chlorethanes [55], and brominated/aromatic compounds [44]. The best studied example for CO_2_-alternative electron acceptors in acetogens is the reduction of the carbon–carbon double bond in phenylacrylates (e.g., caffeate) by the model organism *Acetobacterium woodii* [52, 56, 57].

As shown in this study, *Acetobacterium* spp. appear also to utilize isoprene as an alternative electron acceptor to CO_2_. Isoprene reduction to methylbutenes depended on the presence of H_2_ and CO_2_ [Fig. 4], common substrates for acetogens, which can otherwise only be metabolized by methanogens under anaerobic conditions. Methanogens were not present in isoprene amended enrichment cultures and no methane formation was observed. In cultures resupplied with H_2_, HCO_3_^−^, and isoprene, 40% less acetate was formed compared to H_2_ and HCO_3_^−^ supplied cultures [Fig. 7c] suggesting that the *Acetobacterium* spp. transferred electrons from H_2_ to isoprene at the expense of CO_2_ reduction. In cultures without isoprene, 400 μmoles acetate were produced, which required oxidation of 1600 μmoles H_2_ (Eq. 5) or 3200 μmoles of electrons. However, cultures with isoprene produced only 240 μmoles of acetate requiring 960 μmoles H_2_. In addition 800 μmoles H_2_ were required to reduce 800 μmoles of isoprene. Altogether that totals 1760 μmoles H_2_ or 3520 μmoles of electrons transferred in cultures supplied with isoprene. Since similar amounts of electrons were transferred in cultures with (3520 μmoles) and without isoprene (3200 μmoles) and similar cell densities in each condition were observed, it can be concluded that the shortage of acetate in isoprene amended microcosms results from the reduction of isoprene instead of CO_2_. Isoprene reduction might, therefore, also be coupled to energy conservation. Similar results were found by Hansen et al. [58] in the *A. woodii* NZva16 strain grown on H_2_, CO_2,_ and caffeate, a key intermediate in lignin synthesis in plants. It was shown that 11 mM caffeate was reduced to hydrocaffeate and ~14–40% less acetate was formed compared to cultures without caffeate.

Considering the Δ*G*° values of the isoprene/methylbutene couple (Eq. 4), isoprene reduction is energetically favored over CO_2_ reduction (Eq. 5). This could lead to preferential use of isoprene over CO_2_. However, in the case of caffeate (caffeate/hydrocaffeate), its reduction and acetogenesis from H_2_ and CO_2_ were either catalyzed simultaneously or CO_2_ was preferred over caffeate [56, 58]. Tschech and Pfennig [59] observed that caffeate was preferentially used over CO_2_ as an electron acceptor in the presence of methanol (methanol/caffeate 1:3). In the enrichment culture generated in this study, it remains to be tested whether isoprene reduction also occurs simultaneously catalyzed by a dominating *Acetobacterium* spp. by multiple or if one species only reduces isoprene and not CO_2_.

It was unexpected that pure *Acetobacterium* isolates (*A. woodii* DSM 1030, *A. malicum* DSM 4132, and *A. wieringae* DSM 1911) showed no isoprene reduction activity. However, *Acetobacterium* are a particularly tight phylogenetic group with 96–99% 16S gene sequence similarity between species [60]. It is possible that an uncultured species of *Acetobacterium* is responsible for isoprene reduction, or the gene enabling the reduction was acquired via horizontal gene transfer. These possibilities will be investigated in future studies. Also, *Comamonadaceae* cannot be conclusively excluded from involvement in isoprene reduction as Illumina sequencing data did not reveal the genus of organisms present. There is a possibility that *Hydrogenophaga* lineages, some of which can oxidize H_2_ and fix CO_2_ [61], contribute to isoprene reduction.

Bioenergetics and growth efficiencies of acetogens can be evaluated by using the acetate-to-biomass ratios [62]. Even though less acetate was formed in isoprene amended cultures, similar cell densities were achieved. This suggests that isoprene reduction is coupled to ATP synthesis as shown for caffeate reduction by *A. woodii* [58]. The current model in *A. woodii* suggests that electrons flow from H_2_ to NAD^+^ and ferredoxin, which are reduced by a electron-bifurcating hydrogenase [58]. A Na^+^-translocating ferredoxin: NAD^+^-oxidoreductase then oxidizes ferredoxin (Rnf complex) and generates a Na^+^ gradient over the cytoplasmic membrane for ATP synthesis [57, 63, 64]. It is possible that some of these enzymes might be involved in isoprene reduction. But in contrast to phenylacrylates, fumarate and chlorethenes, isoprene is an unsubstituted alkene making nucleophilic attack more difficult.

### Hydrogenation of isoprene

In the present study, we have shown that isoprene is hydrogenated to three methylbutene isomers with proportions remaining constant throughout the experiments (2-methyl-1-butene 98%, 3-methyl-1-butene 1-2%, and 2-methyl-2-butene < 1%) i.e., fully reduced alkanes were not produced [Supplementary Fig. S2]. Hydrogenation of a double bond is a thermodynamically favorable reaction because it forms a more stable (lower energy) product. The released heat is referred to as the heat of hydrogenation (ΔHh_298_°), which reflects the stability of a molecule. Isoprene, due to its conjugated doubled bonds, has a ΔHh_298_° value of −55 kcal mol^−1^ compared to its reduced forms (ΔHh_298_° 3-methyl-1-butene; −29.9 kcal mol^−1^, 2-methyl-1-butene; −28.24 kcal mol^−1^ and 2-methyl-2-butene; −26.74 kcal mol^−1^ [65]. Therefore, reduction of a single double bond requires more activation energy and a different set of enzymes. This could explain why ethylene (H_2_C = CH_2_) was not reduced to ethane by the active isoprene reducing culture. A few observations of microbial hydrogenation of ethylene to ethane have been made [66–69] but a pure bacterial culture or responsible enzymes have not been identified. It remains to be tested whether the enzymatic hydrogenation of isoprene to methylbutene occurs directly or via an intermediate hydration product (e.g., 3-Methyl-3-buten-1-ol).

### The physiological role of isoprene reduction-metabolic strategies of acetogens

Depletion of electron acceptors creates a niche for acetogens and methanogens due to their ability to obtain energy from CO_2_ reduction via H_2_ oxidation [70, 71]. In these environments acetogens compete with methanogens for H_2_.

6 $$\begin{array}{*{20}{c}} {{\mathrm{HCO}}_3^ - {\mathrm{ + }}4{\mathrm{H}}_2 + {\mathrm{H}}^ + \to {\mathrm{CH}}_4 + {{{\rm{H}}_2{\rm{O}}}}} & {\Delta G^\circ {\mathrm{ = }} - 135\,{\mathrm{kJ}}{\mathrm{/}}{\mathrm{mol}}} \end{array}$$

Thermodynamically, hydrogenotrophic methanogenesis (Eq. 6) is favored over acetogenesis (Eq. 5), therefore acetogens are physiologically less competitive for H_2_ when it is a limiting resource. Yet, their ability to use different electron acceptors enhances the in situ competitiveness of acetogens [72, 73]. Given that Δ*G*° values for hydrogenotrophic isoprene reduction (Eq. 4) and bicarbonate reduction to methane (Eq. 6) are equivalent, the use of isoprene as an alternative electron acceptor would enable acetogens to compete with methanogens at similar H_2_ threshold concentrations.

According to Lever et al. [70] there are two hypotheses for how the wide metabolic spectrum of acetogens enables them to coexist with sulfate reducers and methanogens. Firstly, metabolic versatility leads to niche differentiation with respect to substrate and secondly acetogens can pool energy from a broad range of metabolic reactions (e.g., simultaneous CO_2_/caffeate and now CO_2_/isoprene reduction). Being able to utilize a substrate that inhibits a potential competitor could add a third strategy, whereby environments with isoprene present logically favouring acetogenic organisms.

In this study, isoprene was observed to inhibit methanogenesis. The inhibitory effect of isoprene on methanogenesis in sediment slurries has been observed previously [74] where isoprene (3.6 mM in liquid phase) partially inhibited methanogenesis. Little is known about isoprene concentrations in anaerobic environments and hence it cannot be concluded yet if isoprene mediated inhibition of methanogenesis is ecologically or biogeochemically relevant. Therefore, it is also not yet clear whether isoprene reducers can consume atmospheric isoprene or if they consume isoprene generated in soil sources such as bacteria [20]. Isoprene concentrations used in this and other studies on isoprene biotransformation are in the order of 10^6^ times higher than observed under natural conditions [9, 30, 34, 75–77]. Regardless, even at high concentrations the observed capacity for microbes to consume isoprene is far from saturated, suggesting the isoprene consuming microbial community is large relative to the isoprene supply and/or enzymes involved are highly efficient [75].

## Conclusion

This study explored the anaerobic metabolism of isoprene, which is quantitatively the most abundant volatile hydrocarbon emitted by plants. Isoprene was shown to act as an electron acceptor for homoacetogenic bacteria belonging to the *Acetobacterium* genera and was shown to be reduced to three different methylbutene isomers in an H_2_ dependent manner. Isoprene had an inhibitory effect on methanogenesis so there may be a relationship between isoprene emission and methane biosynthesis. The discovery of biohydrogenation of this unsubstituted, unsaturated alkene whose functional group makes up 60% of all Natural Products (isoprenoids or terpenoids) on Earth [78] warrants further investigation. Future experiments should explore the isoprene reduction mechanism, the enzymes involved and its ecological role in biogenic methane sources. Overall, this research demonstrates that isoprene is capable of being reduced in anaerobic environments, implicating a potential previously undiscovered isoprene sink.

## Supplementary information

Supplementary material
