# Supplementary material for: Reductive metabolism of the important atmospheric gas isoprene by homoacetogens
Source: ISME J. 2019 Jan 14;13(5):1168–82. doi: 10.1038/s41396-018-0338-z (PMC6474224; doi:10.1038/s41396-018-0338-z)
Supplement: Supplementary file 1 — Supplementary material [file 41396_2018_338_MOESM1_ESM.docx]

**

**

**Figure S1.** Massspectra of 2-metyhl-1-butene (A) and 3-methyl-1-butene (B) derived from isoprene reducing cultures.

**
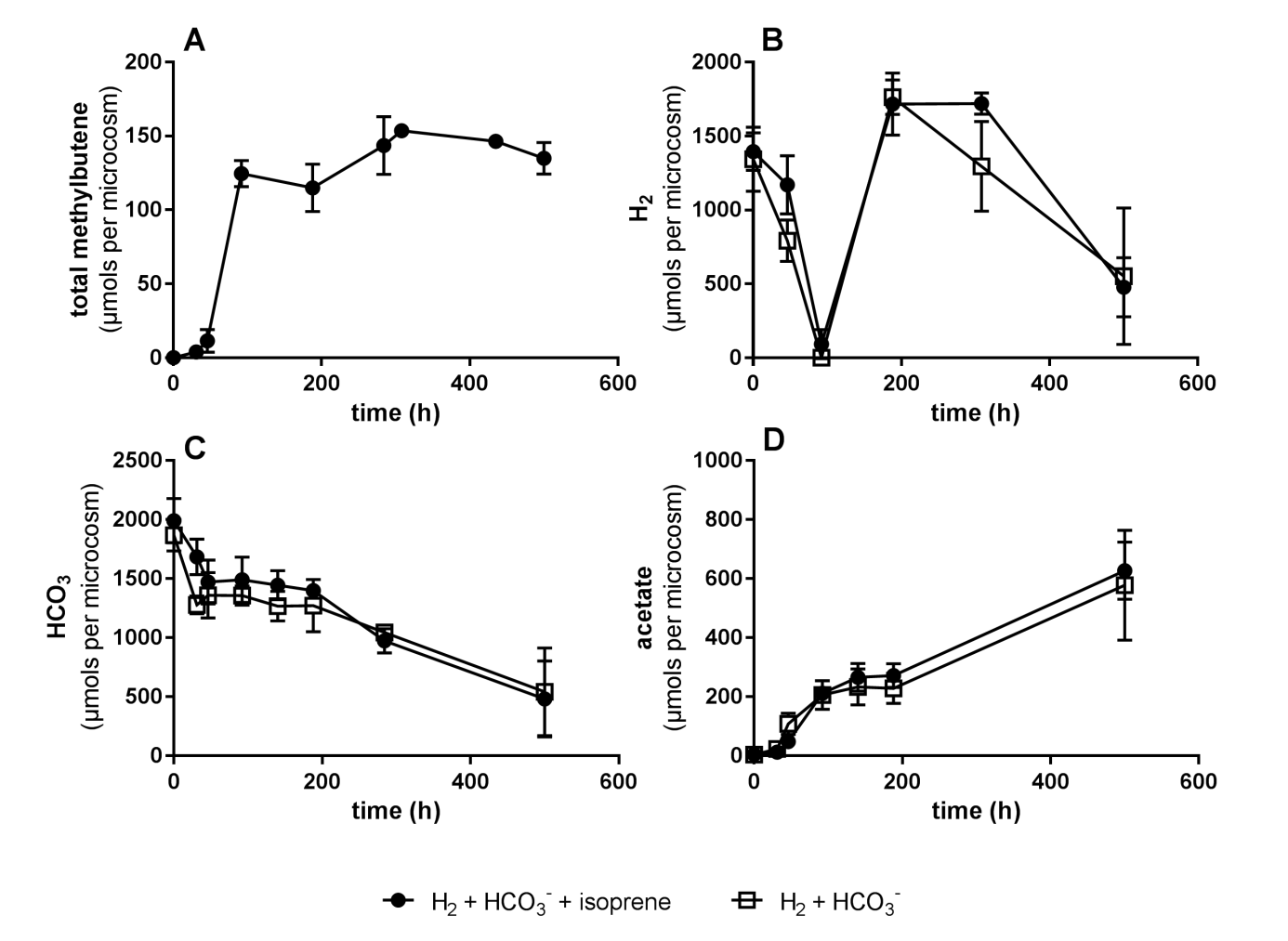
**

**Figure S2.** Methylbutene amounts of highly enriched isoprene reducing culture after the addition of new H_2_ after 200 h (A). Consumption of H_2_ (B) and HCO_3_^-^ (C) and acetate formation (D) in anaerobic cultures. Error bars represent one standard deviation (n = 4).


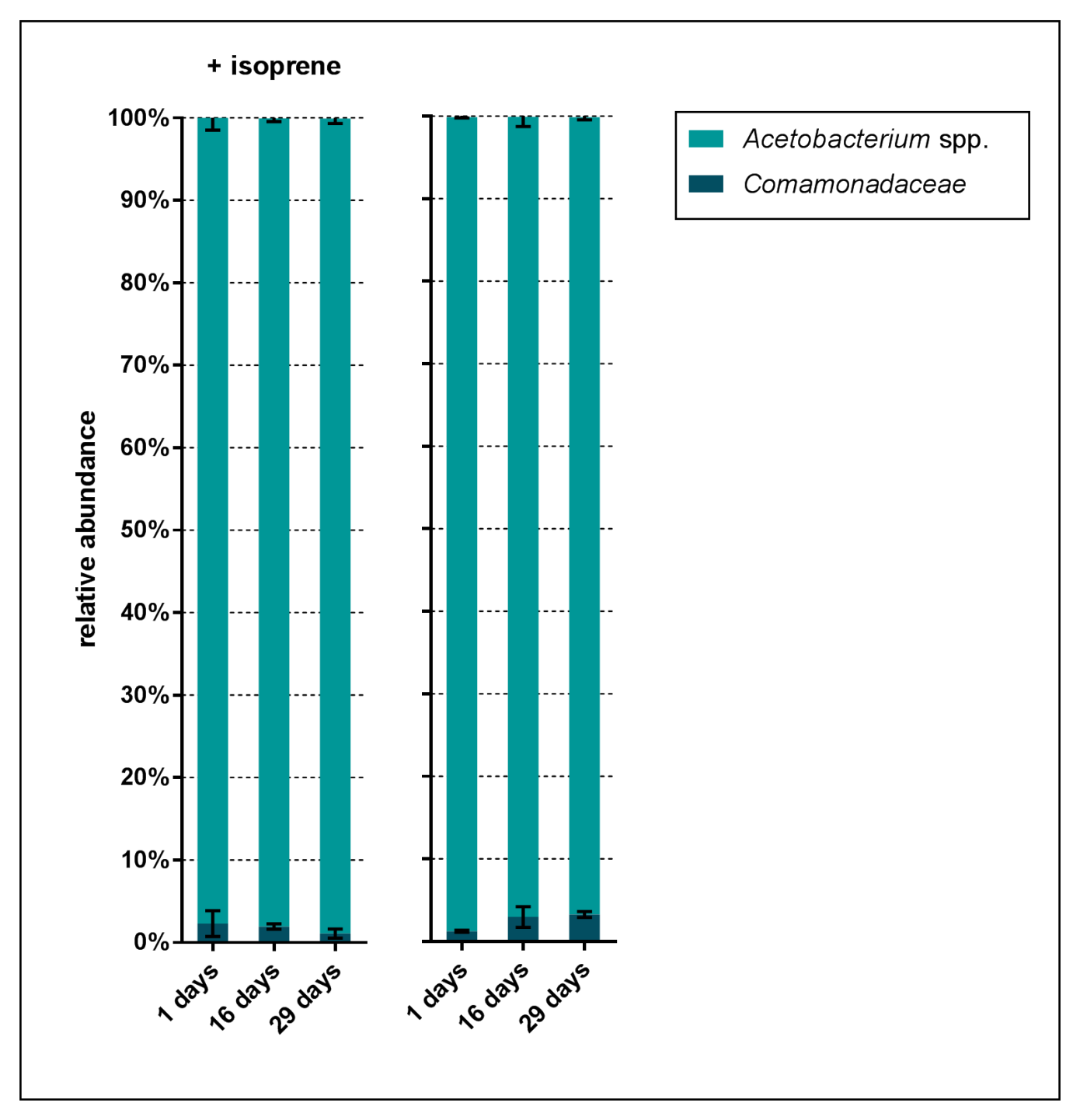


**Figure S3.** Composition of the bacterial populations at Family and if possible on Genus level (based on 16S rDNA illumina sequencing) of an isoprene reducing, continuous grown batch culture on H_2_ + HCO_3_^-^ + isoprene and a H_2_ + HCO_3_^-^ control at different time points 1, 16 and 29 days. In both set ups *Acetobacterium* was found most abundant. *Comamonadaceae* are also present in smaller numbers (0.8 % - 4 %). Error bars represent one standard deviation (n = 3).


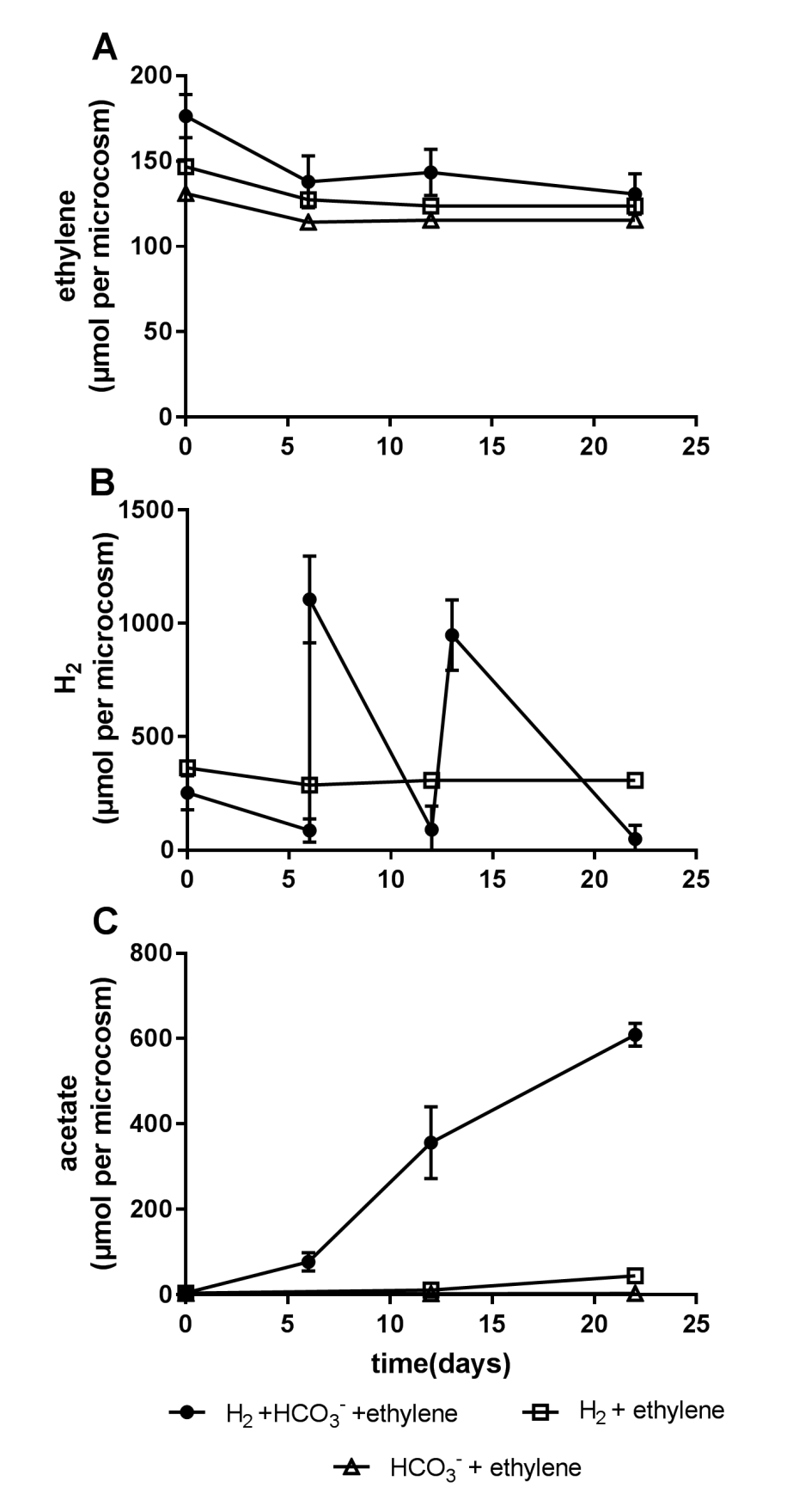


**Figure S4.** Incubation of the active isoprene reducing culture with 160 µmoles of ethylene, H_2_ and HCO_3_^-^. Ethylene concentration stayed stable over time H_2_ + HCO_3_^-^ + ethylene and the controls (A). H_2_ was depleted and acetate was formed respectively (**B &C**). Error bars represent one standard deviation (n = 4).


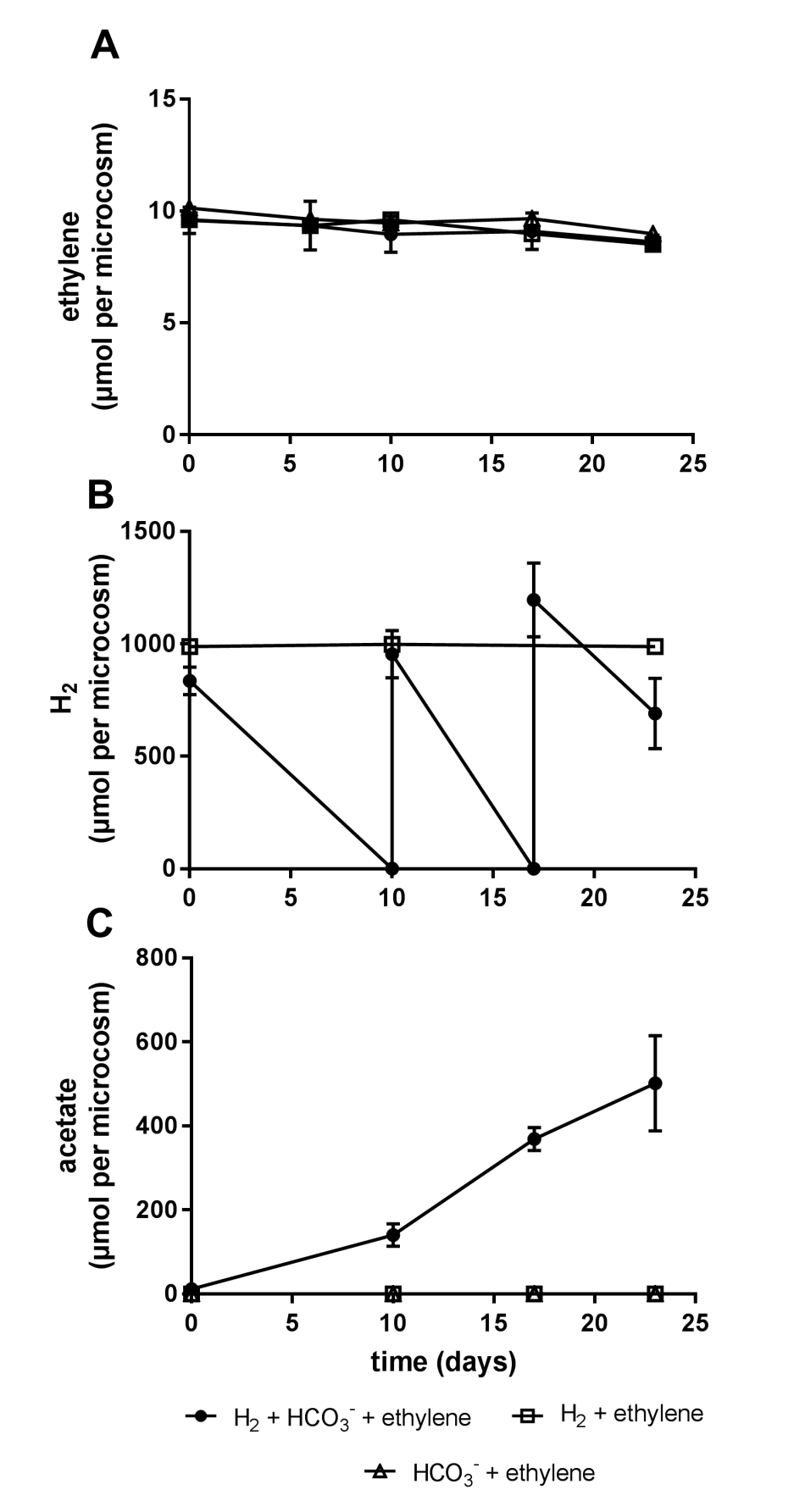


**Figure S5.** Incubation of the active isoprene reducing culture with 10 µmoles of ethylene, H_2_ and HCO_3_^-^. Ethylene concentration stayed stable over time H_2_ + HCO_3_^-^ + ethylene and the controls (A). H_2_ was depleted and acetate was formed respectively (**B &C**). Error bars represent one standard deviation (n = 4).
